# HPV-associated differential regulation of tumor metabolism in oropharyngeal head and neck cancer

**DOI:** 10.18632/oncotarget.17887

**Published:** 2017-05-16

**Authors:** Young-Suk Jung, Abdo J. Najy, Wei Huang, Seema Sethi, Michael Snyder, Wael Sakr, Gregory Dyson, Maik Hüttemann, Icksoo Lee, Rouba Ali-Fehmi, Silvia Franceschi, Linda Struijk, Harold E. Kim, Ikuko Kato, Hyeong-Reh Choi Kim

**Affiliations:** ^1^ Department of Pathology, Barbara Ann Karmanos Cancer Institute, Wayne State University School of Medicine, Detroit, MI, USA; ^2^ Department of Oncology, Barbara Ann Karmanos Cancer Institute, Wayne State University School of Medicine, Detroit, MI, USA; ^3^ Division of Radiation Oncology, Barbara Ann Karmanos Cancer Institute, Wayne State University School of Medicine, Detroit, MI, USA; ^4^ Center for Molecular Medicine and Genetics, Wayne State University School of Medicine, Detroit, MI, USA; ^5^ International Agency for Research on Cancer, Lyon, France; ^6^ DDL Diagnostic Laboratory, Rijswijk, The Netherlands; ^7^ Current Address: Pusan National University College of Pharmacy, Geumjeong-gu, Busan, Republic of Korea; ^8^ Current Address: College of Medicine, Dankook University, Cheonan-si, Chungcheongnam-do, Republic of Korea

**Keywords:** head and neck squamous cell carcinoma, human papillomavirus, radiation, glucose metabolism, mitochondrial respiration

## Abstract

HPV-positive oropharyngeal cancer patients experience significantly lower locoregional recurrence and higher overall survival in comparison with HPV-negative patients, especially among those who received radiation therapy. The goal of the present study is to investigate the molecular mechanisms underlying the differential radiation sensitivity between HPV-negative and HPV-positive head and neck squamous cell carcinoma (HNSCC). Here, we show that HPV-negative HNSCC cells exhibit increased glucose metabolism as evidenced by increased production of lactate, while HPV-positive HNSCC cells effectively utilize mitochondrial respiration as evidenced by increased oxygen consumption. HPV-negative cells express HIF1α and its downstream mediators of glucose metabolism such as hexokinase II (HKII) and carbonic anhydrase IX (CAIX) at higher levels, while the expression level of cytochrome *c* oxidase (COX) was noticeably higher in HPV-positive HNSCC. In addition, the expression levels of pyruvate dehydrogenase kinases (PDKs), which inhibit pyruvate dehydrogenase activity, thereby preventing entry of pyruvate into the mitochondrial tricarboxylic acid (TCA) cycle, were much higher in HPV-negative HNSCC compared to those in HPV-positive cells. Importantly, a PDK inhibitor, dichloroacetate, effectively sensitized HPV-negative cells to irradiation. Lastly, we found positive interactions between tonsil location and HPV positivity for COX intensity and COX/HKII index ratio as determined by immunohistochemical analysis. Overall survival of patients with HNSCC at the tonsil was significantly improved with an increased COX expression. Taken together, the present study provides molecular insights into the mechanistic basis for the differential responses to radiotherapy between HPV-driven *vs*. spontaneous or chemically induced oropharyngeal cancer.

## INTRODUCTION

Head and neck squamous cell carcinoma (HNSCC) is among the 10 most common cancers worldwide. In the USA, despite the overall declining trend in the incidence of HNSCC, reflecting the decreasing trend in tobacco consumption, the incidence of HNSCC from the oropharyngeal sites, especially the tonsil and the base of the tongue, is on the rise [1]. This rising incidence is attributable to human papillomavirus (HPV) [2, 3] and the oropharynx now represents the most common site of HPV-associated cancers surpassing cervical cancer [4].

One of the most intriguing clinical characteristics of HPV-associated HNSCC is better survival compared with HPV-negative HNSCC [5–12]; thus, HPV positivity serves as an independent prognostic factor in HNSCC patients. These studies report that the risk of overall death is halved when the tumor is HPV16 positive. Remarkably, this survival advantage has been attributed to better responses and higher sensitivity to radiotherapy or radiochemotherapy [6, 7, 11]. One study reports that the response to radiation is 4 times better in HPV (+) cancer [6], and others have demonstrated that improved survival is limited to patients who underwent radiotherapy with a hazard ratio of death or recurrence of 0.2-0.25 [7, 11].

One important biological factor that substantially affects the risk of tumor-related death in patients with head and neck cancer is tumor hypoxia [13–15]. Cancer cells evolve several alterations in their metabolism to survive in unfavorable microenvironments, while retaining their ability to proliferate [16]. A classical metabolic adaptation of tumor cells is a shift to glycolysis as a main source of ATP, rather than oxidative phosphorylation (OXPHOS), irrespective of oxygen availability, a phenomenon referred to as the Warburg effect [17]. This phenotype, associated with induction of the hypoxia-inducible factor 1 (HIF1) pathway, promotes cell survival, the generation of biosynthetic precursors for proliferation, and invasiveness [16, 18, 19]. Indeed, HIF1α has been identified as a poor prognostic factor in patients with head and neck squamous cell carcinoma treated with radiotherapy [20]. HIF1α regulates the transcription of many genes involved in cancer pathogenesis and progression to more aggressive phenotypes [21, 22]. Of them, HIF1α upregulates the transcription of genes encoding glycolytic enzymes and glucose transporters, resulting in increased glycolysis [21, 22]. In addition, HIF1α upregulates the pyruvate dehydrogenase kinases (PDKs) that inhibit pyruvate dehydrogenase (PDH), thereby preventing entry of pyruvate into the mitochondrial tricarboxylic acid (TCA) cycle. This reduction in the flow of glucose-derived pyruvate into the TCA cycle reinforces anaerobic glycolysis and spares oxygen consumption, promoting the glycolytic phenotypes.

The goal of the present study is to investigate the molecular characteristics underlying the differential radiation sensitivity between HPV(-) and HPV(+) HNSCC, with a particular focus on the HIF1α pathway and tumor metabolism using cell lines and tissue sample from HNSCC patients whose HPV status was known. Here we show that HPV (-) cells express higher levels of HIF1α, hexokinase II (HKII) and PDKs, while HPV(+) cells express higher levels of cytochrome *c* oxidase (COX). Accordingly, HPV (-) cells exhibited a higher rate of glycolysis while HPV (+) cells displayed higher oxygen consumption. Consistently with previous reports, HPV (-) cells exhibited the radiation-resistant phenotype compared to HPV (+) cells. Importantly, when PDK activity was inhibited, HPV (-) cells became sensitive to radiation. Moreover, when we correlated overall survival in 79 oropharyngeal patients with COX and HKII expression levels, and overall survival for tonsil cancer was significantly improved with increased COX expression or COX/HKII ratio level. These findings provide insights into the mechanistic basis for the differential responses to radiotherapy and survival between HPV-driven *vs*. spontaneous or chemically-induced oropharynx.

## RESULTS

### Differential regulation of the HIF1α and tumor metabolism pathways between HPV-positive and -negative HNSCC

**Figure 1:**
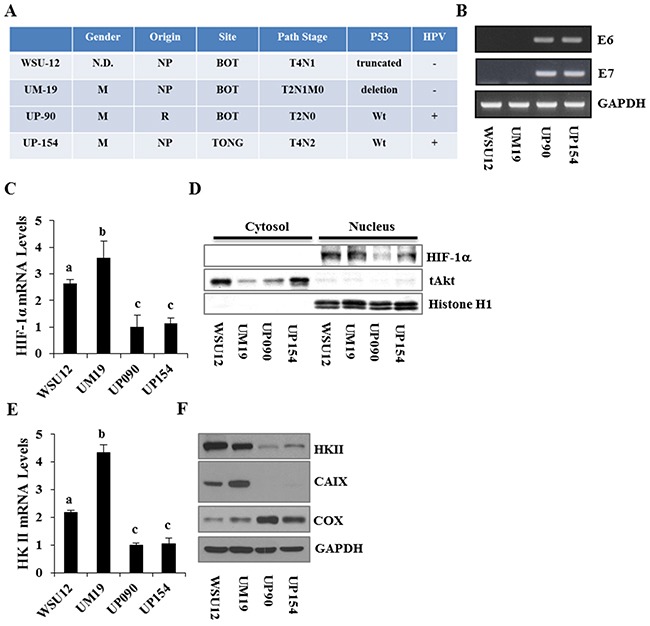
Characterization and differential expression of metabolism regulators between HPV-negative and HPV-positive HNSCC cell lines **(A)** Clinical characteristics of the HNSCC cell lines. **(B)** RT-PCR analysis of HPV E6 and E7 expression in HNSCC cell lines. **(C)** Quantitative RT-PCR analysis of HIF1α mRNA. **(D)** Immunoblot analysis of cytoplasmic and nuclear HIF1α proteins in HNSCC cell lines. **(E)** Quantitative RT-PCR analysis of Hexokinase II. **(F)** Immunoblot analysis of hexokinase II, CAIX and cytochrome *c* oxidase subunit 1. Each bar represents the mean ± S.D. Means with different letters (a, b, c) are significantly different from one another at *P* value < 0.05 (ANOVA followed by Newman-Keuls test).

To examine whether regulation of HIF1α and/or glucose metabolism in HNSCC cells are associated with the HPV status, we utilized two HPV (-) HNSCC cell lines, WSU12 and UM19, and two HPV-positive HNSCC cell lines, UP90 and UP154. Tumor site, status of the tumor suppressor p53 and clinical information on patients from whom these cell lines were established are summarized in Figure 1A. RT-PCR analysis of the HPV oncogenes E6 and E7 confirmed the HPV status in these cell lines (Figure 1B). First, we determined the expression levels and subcellular localization of HIF1α, a signaling molecule/transcription factor known as a poor prognostic factor in patients with HNSCC [20]. Quantitative RT-PCR analysis showed that HPV (-) HNSCC cells express higher levels of HIF1α mRNA compared to HPV (+) HNSCC cells (Figure 1C). The levels of nuclear HIF1α proteins were markedly higher in HPV (-) cells (Figure 1D). Among target genes of HIF1α, we found that the levels of hexokinase II (HKII, the rate limiting enzyme in glucose metabolism) and carbonic anhydrase IX (CAIX, an enzyme that hydrates metabolically released CO_2_ into H^+^ and HCO_3_) were higher in HPV(-) cells compared to HPV(+) cells Figure 1E&1F). In contrast, the expression level of cytochrome *c* oxidase (COX), the last enzyme protein complex (complex IV) in the mitochondrial electron transport chain, was noticeably higher in HPV (+) HNSCC. Interestingly, the expression level of COX was inversely correlated with the expression levels of HKII in HNSCC cells (Figure 1F).

**Figure 2:**
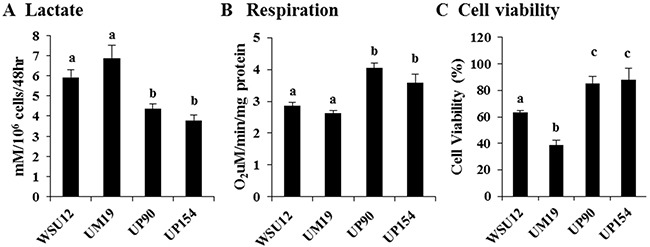
Increased glycolysis in HPV-negative cells and increased respiration in HPV-positive HNSCC **(A)** Lactate production, **(B)** mitochondrial respiration and **(C)** cell viability in the absence of glucose in HNSCC cell lines. Each bar represents the mean ± S.D. Means with different letters (a, b, c) are significantly different from one another at *P* value < 0.05 (ANOVA followed by Newman-Keuls test).

The above results suggest that there might be differential regulation of anaerobic glucose metabolism and mitochondrial oxidative phosphorylation in HPV (-) vs. HPV (+) HNSCC cells. To examine this, we measured the levels of lactate (the end product of anaerobic glycolysis) and oxygen consumption in these cells. As predicted by gene expression profiles, HPV (-) HNSCC cells exhibited increased production of lactate with reduced oxygen consumption, whereas HPV (+) cells showed the inverse relationship (Figure 2A&2B). Upon glucose deprivation for 48 hours, 40-60% HPV (-) HNSCC cells died whereas ~90% HPV (+) cells survived (Figure 2C). These results showed that spontaneously/chemically induced HPV (-) HNSCC cells exhibit pronounced glucose metabolism and that their survival depends on glucose supply.

### *In vivo* validation of differential expression of tumor metabolism markers in oropharyngeal cancer patients

Next, we examined the association between the HPV status and tumor metabolism markers (HKII and COX) using 79 HNSCC patient samples with which we previously determined the presence of HPV DNA and its genotype [11]. All but two cases were positive for COX and all were positive for HKII staining, as expected since these are essential metabolic enzymes. There were marginally significant associations between poor tumor differentiation and higher COX intensity (P=0.092), between strong HKII intensity and regional/distal stage (p=0.081) and between HKII dominant COX/HKII index ratio and smoking at diagnosis (p=0.059).

**Figure 3:**
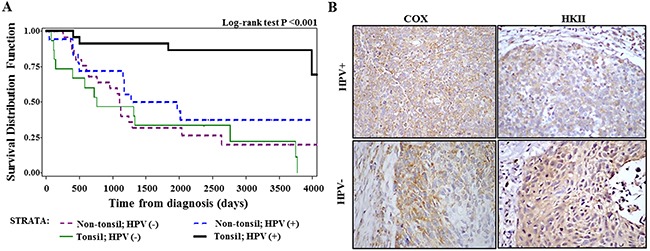
Analysis of tissue samples from patients with HNSCC **(A)** Overall survival of oropharyngeal cancer patients stratified by tonsil location and HPV status. **(B)** Immunohistochemical analysis of cytochrome *c* oxidase (COX) subunit 1 and hexokinase II (HKII) in HPV-positive and HPV-negative tissues samples from patients with oropharyngeal cancer (x400 magnification).

Since increased overall survival associated with HPV-positivity was primarily seen for tonsil cancer but not for other oropharyngeal sites in our samples (Figure 3A), we stratified further analyses between tonsil and non-tonsil groups. At the tonsils (N=36), HPV-positivity was marginally significantly associated with the COX/HKII index ratio (p=0.052) and with moderate-strong COX intensity (P=0.081) among non-smokers only (N=15), while no associations were present in current smokers at diagnosis (n=21). As shown in Table 1A, the ordinal logistic regression analyses with three response levels of COX intensity (none-weak, moderate, strong) and index ratio (HKII dominant, even, COX dominant) indicate there were inverse interactions between HPV and smoking status with a statistically significant interaction term for COX intensity (P=0.036). Taking this interaction into consideration, the regression coefficient for HPV positivity was statistically significant or marginally statistically significant for COX intensity (P=0.045) and COX/HKII index ratio (P=0.099). There were no associations with these parameters at non-tonsil site. Importantly, we found positive interactions between tonsil location and HPV positivity for these markers, (p=0.067 for COX intensity and P= 0.045 for COX/HKII index ratio). These analyses also demonstrated that the proportional odds assumption was met for COX/HKII index ratio at both tonsil and non-tonsil sites and for COX intensity at non-tonsil site but not met at tonsil site (P=0.007). Thus, we further analyzed COX intensity for two separate cutoff points by using binary logistic regression models. As shown in Table 1B, the regression coefficients for HPV and its interaction with smoking were in the same direction at tonsil site with different cutoff points, but their associations were much stronger for ‘moderate + strong’ than the ‘strong’ cutoff point, suggesting that the results seen in the ordinal logistic regression models were primarily driven by the outcome of moderate and strong staining combined. Representative images of COX and HKII staining in HPV (+) and HPV (-) tonsil cancer specimen are shown in Figure 3B.

**Table 1A:** Results of ordinal logistic regression analyses for response variable of COX intensity and COX/HKII index ratio in three levels by tumor location

			Tonsil (N=36)			Non-tonsil (N=43)		P-value forinteractionwith tonsil
Response variables	Explanatory variables	Regression coefficient	Standard Error	P-values	Regression coefficient	Standard Error	P-values	
Cox intensity	HPV positive	5.358	2.670	0.045	0.143	1.245	0.909	0.067
	Smoking	1.427	1.266	0.259	0.642	0.552	0.245	0.548
	HPV *smoking	-3.162	1.511	0.036	-0.707	0.851	0.406	0.137
CoX/HKII index ratio	HPV positive	4.738	2.868	0.099	-1.255	1.262	0.320	0.045
	Smoking	0.630	1.374	0.647	0.000	0.531	1.000	0.650
	HPV*smoking	-2.582	1.608	0.108	0.486	0.849	0.568	0.076

OR: Odds ratio

P-values for interaction with tonsil were derived from a separate model including both sites

Non tonsil cases were 42 for Cox/HKII index ratio

**Table 1B:** Results of binary logistic regression analyses for COX intensity with 2 different cutoff points

COX intensity cutoff			Tonsil (N=36)			Non-tonsil (N=43)		P-value forinteractionwith tonsil
	Explanatory variables	Regression coefficient	Standard Error	P-values	Regression coefficient	Standard Error	P-values	
None-weak vs moderate-strong	HPV positive	7.300	3.333	0.029	0.843	1.533	0.766	0.078
	Smoking	2.303	1.449	0.112	1.037	0.738	0.582	0.436
	HPV *smoking	-4.349	1.881	0.021	-0.616	1.179	0.160	0.093
None-moderate vs strong	HPV positive	1.666	2.791	0.551	-0.462	1.405	0.742	0.496
	Smoking	-0.406	1.394	0.771	0.250	0.576	0.665	0.664
	HPV*smoking	-1.050	1.631	0.520	-0.702	1.010	0.487	0.856

OR: Odds ratio

P-values for interaction with tonsil were derived from a separate model including both sites

We further analyzed overall survival of the 79 oropharyngeal patients according to COX or COX/HKII index ratio, adjusted for tonsil subsite and subsite-marker interactions, using stratified analyses by radiation treatment. Overall survival at the tonsil was significantly improved with an increase in COX expression level (HR =0.42, 95% CI 0.18-0.98, per one level increase) and in COX/HKII ratio (HR 0.37, 95%CI 0.17-0.81, per one level increase). Simultaneous adjustment for HPV-positivity and smoking status weakened these associations (P=0.06-0.15).

### Tumor metabolism regulation and radiosensitivity

**Figure 4:**
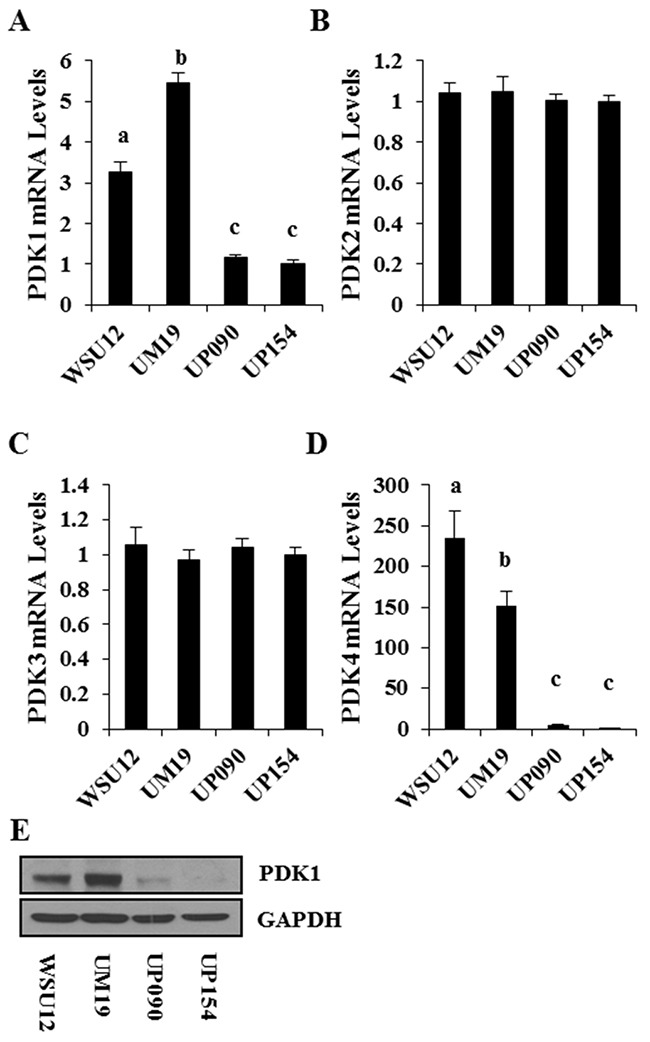
PDK expression profile in HNSCC cell lines Quantitative RT-PCR of PDK1 **(A)**, PDK2 **(B)**, PDK3 **(C)**, and PDK4 **(D)**. Each bar represents the mean ± S.D. Means with different letters (a, b, c) are significantly different from one another at *P* value < 0.05 (ANOVA followed by Newman-Keuls test). **(E)** Immunoblot analysis of PDK1.

Based on the above results, we hypothesized that HIF1α-induced glucose metabolism is associated with radioresistant phenotype in HPV (-) HNSCC cells. Here, we wished to identify tumor metabolism regulator(s) that are differentially expressed between HPV (+) and HPV (-) cells and targetable for the metabolic switch and subsequent regulation of radio-sensitivity. Previous studies suggested that HIF1α regulates an important junction between mitochondrial respiration and glycolysis via upregulation of PDK [23] which is an endogenous inhibitor of PDH that converts pyruvate into acetyl-CoA, the primary fuel for the mitochondrial TCA cycle [24]. Interestingly, quantitative RT-PCR analysis showed that PDK1 and PDK4 were highly expressed in HPV (-) cells compared to HPV (+) cells, while expression levels of PDK2 and PDK3 were comparable (Figure 4A-4D). Immunoblot analysis confirmed the higher expression of PDK1 in the HPV (-) WSU12 and UM19 cell lines (Figure 4E). It should be noted that among the four PDK family members, PDK1 was shown to play a role in glucose metabolism in HNSCC cells [25, 26].

**Figure 5:**
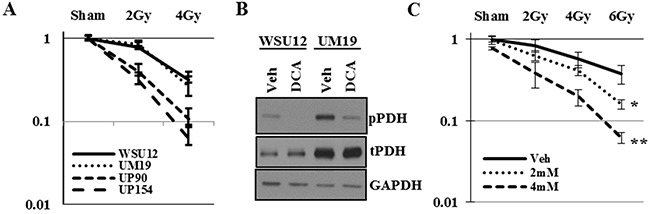
Inhibition of PDK sensitizes HPV (-) HNSCC to radiation **(A)** Clonogenic cell survival assay of HPV (+) and HPV (-) HNSCC upon irradiation. **(B)** HPV(-) WSU12 and UM19 were treated with vehicle (veh) or 12mM DCA and PDK activity was assessed through pyruvate dehydrogenase (PDH) phospohorylation. **(C)** Clonogenic cell survival assay of WSU12 cells upon irradiation with or without DCA treatments. Means with * or ** are significantly different from one another at P value < 0.05 (ANOVA followed by Newman-Keuls test)

Consistent with previous reports, HPV (+) UP90 and UP154 cell lines were more sensitive to radiation treatment as compared to the HPV (-) WSU12 and UM19 cells (Figure 5A). To determine whether inhibition of PDK can sensitize HPV (-) cells to radiation, WSU12 and UM19 cells were treated with the PDK inhibitor dichloroacetate (DCA) followed by irradiation. Inhibition of PDK activity was confirmed by reduced phosphorylation of its substrate PDH (Figure 5B). Clonogenic cell survival assay showed significant sensitization of the WSU12 cell line to radiation treatment in the presence of DCA (Figure 5C). A long-term treatment with DCA was too toxic to UM19 cells; as a result we could not obtain reliable colony formation data using this cell line.

## DISCUSSION

HPV-positive HNSCCs represent a distinct biological entity, often associated with increased expression of p16 in the absence of p53 loss or mutations [27]. This is ascribed to HPV oncoproteins E6 and E7 that interferes with the actions of tumor suppressors p53 and pRb, respectively [28]. Studies have shown that mutant p53 proteins upregulate the HIF1α pathway, resulting in increased glycolysis, angiogenesis and fibrosis, which contribute to the progression of cancer, i.e., local invasion and distant metastasis [29–33]. Increased HIF1α and other hypoxia-responsive genes have been associated with increased risk of treatment failure, recurrence, and reduced overall and disease-free survival [20, 34–37]. Interestingly, a recent study showed that E6 increases the protein stability of HIF1α through hindering the association of the von Hippel-Lindau tumor suppressor gene (VHL)-containing E3 ligase with its target HIF1α, resulting in induction of glycolysis in cervical cancer cells [38]. These *in vitro* mechanistic studies may predict association between HPV and HIF1α-mediated radiotherapy resistance. Contrarily, HPV positivity serves as an independent prognostic factor for higher sensitivity to radiotherapy in patients with HNSCC from the oropharyngeal sites, especially the tonsil and the base of the tongue [6, 7, 11]. Interestingly, clinical studies demonstrated that HIF-1α is a poor prognostic factor in patients with HPV (-) HNSCC treated with radiotherapy [20]. These studies suggest differential involvement of HIF1α between viral- and chemical (tobacco, alcohol)-induced carcinogenesis of HNSCC.

The present study demonstrated that the HIF1α pathway and tumor metabolism are differentially regulated between HPV (+) and HPV (-) HNSCC cells. HPV-positive HNSCC cells depend on mitochondrial respiration with decreased glucose metabolism, whereas HPV-negative and p53 mutated HNSCC cells heavily relied on glycolytic pathways for their survival and that the former group of cells were more radiosensitive than the latter group. Furthermore, we showed that pharmacological manipulation to reverse the glycolytic phenotype in HPV-negative cells increased radiosensitivity. The data from clinical samples also corroborated that COX, the proposed rate-limiting enzyme in mitochondrial respiration pathway [39], was over-expressed in HPV-positive tonsil cancer from non-smokers and that relative expression of a key glycolytic pathway enzyme was decreased in those cancer tissues. Upregulation of mitochondrial respiration pathway, which was assessed by IHC of COX in clinical samples, was also associated with improved overall survival for tonsil cancer, but not for non-tonsil oropharyngeal cancer. There were limitations in our clinical study due to a small sample size, especially when the samples were divided by anatomic subsite and stratified by smoking status, which led to wide confidence intervals of the estimates. Without the interaction terms, there were no significant main effects of HPV or smoking on either a priori planned IHC marker, Thus, the data should be interpreted with caution and need verification with a larger sample.

The scientific premise of the present study is that we may be able to identify new druggable targets for the improvement of radiotherapy outcomes by understanding the molecular mechanisms underlying differential radiation sensitivity between HPV-induced and chemical- induced HNSCC cells. Here, we showed that HPV (-) HNSCC utilizes glycolysis at a much higher level compared to HPV (+) HNSCC. The change in energy metabolism from mitochondrial respiration to anaerobic glycolysis is considered a hallmark of cancer in order to meet increased energy need and biosynthetic intermediates for rapid cell proliferation [40, 41]. Thus, PET scans that map glucose uptake are routinely used in clinical oncology. Recently, it was confirmed that glycolysis was the dominant bioenergetics pathway in HNSCC cells [42]. Glycolysis offers cancer cells a survival advantage independent of oxygen supply and the ability to detoxify chemotherapeutic drugs and reactive free radicals [41]. In addition, products of the glycolytic pathway such as lactate and pyruvate were shown to confer radioresistance and to promote tumor progression [43, 44]. Pharmacological inhibition of this pathway has been shown to inhibit cell proliferation and to enhance radiosensitivity in cells with high rates of glucose usage and glycolysis [42, 45, 46], which is consistent with the results of our experiments using DCA for HPV-negative cells.

In conclusion, the present study provided evidence that metabolic activity is a determinant of oropharyngeal cancer cell response to radiation and identified potential prognostic markers that may help further stratify patient outcome. Our findings may also contribute to the development of mechanism-based radio-sensitizers for de-escalation of radiation dose for HPV-positive oropharyngeal cancers or for more effective treatment for high risk patients with HPV-negative tumors and a smoking history.

## MATERIALS AND METHODS

### Reagents and Antibodies

Anti-β-actin antibody (Ab) was purchased from Sigma (St. Louis, MO); anti-Hexokinase II, total Akt (t-Akt), and anti-PDK1 Abs were from Cell Signaling Technology (Beverly, MA); anti-HIF1α Ab was acquired from NOVUS biologicals (Littleton, CO); anti-COX subunit 1 Ab was obtained from MitoSciences (Eugene, OR); anti-phospho-PDH (pPDH), anti-total PDH and CAIX Ab was from AbCam (Boston, MA).

### Cell culture

Human HNSCC cell lines UP-SCC-090 and UP-SCC-154 (established from specimens of HNSCC patients at University of Pittsburg) and UM-SCC-19 (established at University of Michigan) were grown in DMEM with 10% FBS in a humidified atmosphere of 5% CO_2_ at 37°C. WSU-HN-12 (established in our institute) were maintained in the RPMI1640 with 10% FBS in a humidified atmosphere of 5% CO_2_ at 37°C. Cell lines were obtained in 2011 from the University of Pittsburg (UP-SCC-090 and UP-SCC-154) and the University of Michigan (UM-SCC-19).

### Semi-quantitative RT-PCR

mRNA was isolated from cells using the RNeasy kit (Qiagen). cDNA synthesis was performed with iScript^TM^ cDNA Synthesis Kit (Bio-Rad) followed by PCR using GoTaq Flexi DNA Polymerase (Promega, Madison, WI, USA). Forward and reverse primers specific to human papillomavirus type 16 used in this study are as follows: E6: 5’- ACCCACAGGAGCGACCCAGA -3’, 5’- ACCGGTCCACCGACCCCTTA -3’; E7: 5’- TGAAATAG ATGGTCCAGCTGG -3’, 5’- TGCCCATTAACAGGTCT TCC -3’

### Quantitative RT-PCR

RT-PCR was performed using QPCR SYBR Green Low ROX Mix (Thermo Fisher Scientific Inc.) according to the manufacturer's protocol. Relative values of gene expression were normalized to 18S and calculated using the 2^-ΔΔCt^ method, where ΔΔCt = (ΔCt_target gene_ − ΔCt_18S_)_sample_ − (ΔCt_target gene_ − ΔCt_18S_)_control_. The fold change in relative expression was then determined by calculating 2^-ΔΔCt^. The sequences of human primers for HIF1α, HexokinaseII (HKII), PDK1-4, β-actin, and 18S are HIF1α: 5’- ACCCACCGCTGAAACGCCAA-3’, 5’- TCAGGGCTTG CGGAACTGCT-3’; HKII: 5’- AGGCGATGAGGGGCG GATGT-3’, 5’- CTGGCGGGCACGGTGTTGAT-3’; PDK1: 5’- ATTCAAGTTCATGTCACGCTGG-3’, 5’- TTTCCTCAAAGGAACGCCAC-3’; PDK2: 5'- AGGAC ACCTACGGCGATGA-3', 5'- TGCCGATGTGTTTGGG ATGG-3'; PDK3: 5'- GCCAAAGCGCCAGACAAAC-3', 5'- CAACTGTCGCTCTCATTGAGT-3'; PDK4: 5'- TTAT ACATACTCCACTGCACCA-3', 5'- ATAGACTCAGAA GACAAAGCCT-3'; β-actin: 5’- CTCACCGAGCGCGG CTACA-3’, 5’- CTCCTGCTTGCTGATCCACAT-3’; 18S: 5’- CGGCGACGACCCATTCGAAC-3’, 5’- GAATC GAACCCTGATTCCCCGTC-3’.

### Lactate production

Conditioned media were collected and the level of lactate was measured using the lactate colorimetric assay kit II according to the manufacturer's instructions (BioVision Research Products, Mountain View, CA). Two separate experiments were performed in triplicate (a total of 6 measurements per condition).

### Oxygen consumption

Oxygen consumption of the cells was measured in a closed 200 μl chamber equipped with a micro Clark-type oxygen electrode (Oxygraph system, Hansatech) at 25°C and analyzed with Oxygraph software. 500 μM KCN was added at the end of the each measurement to inhibit cytochrome *c* oxidase. Non-cytochrome *c* oxidase-based consumption was subtracted to determine mitochondrial respiration rates. Cell lysates were collected and protein concentration was determined with the DC protein assay kit (Bio-Rad). The respiration rate was deduced based on the level of oxygen consumption (μM/min per mg protein).

### Cell survival upon glucose deprivation

Cell number was determined by an indirect colorimetric assay (MTT assay). In brief, cells were plated in a 96-well culture plate (4×10^3^ cells/well) overnight followed by culture in glucose-free and serum-free medium for 48 h. MTT (0.5 mg/ml) was then added, and the plates were incubated for 4 h at 37 °C. The formed formazan was extracted with acidic isopropanol, and the absorbance of the converted dye was measured at a wavelength of 570 nm, with background subtraction at 650 nm, using a Benchmark microplate reader (Bio-Rad).

### Immunohistochemical (IHC) analysis of HNSCC patient specimen

Details concerning the patient study using archived clinical samples are described elsewhere [11]. Briefly, patients with squamous cell carcinoma (SCC) of the mouth, pharynx, nose, or larynx who had surgical resection at Wayne State University affiliated hospitals were identified through a population-based cancer registry, the Metropolitan Detroit Cancer Surveillance System (MDCSS). Formalin fixed paraffin embedded tissue blocks were used to determine the presence of HPV DNA and its genotype using a sensitive broad-spectrum PCR technique the SPF_10_ PCR-DEIA-LiPA_25_ version 1 method (Labo Biomedical Products, Rijswijk, The Netherlands) [47, 48]. Patients’ demographics, tumor characteristics and vital status were obtained through record linkage with MDCSS and information regarding smoking and alcohol consumption was abstracted from medical record [49]. Primary sites of cancer were grouped into oropharyngeal or other sites based on International Classification of Diseases for Oncology (ICDO) 4 digit topology codes. Eighty one cases from the oropharyngeal site, defined as C019-C020 (base and dorsal surface of tongue), C051 (soft palate), C052 (uvula), C090-C103 and C108-C109 (all tonsil sites and all oropharynx sites except branchial cleft), were included in this study and their tumor sections were examined for COX subunit 1 and HKII protein expression by IHC. Antigen retrieval was performed in sodium citrate buffer and tissue slides were incubated overnight at 4°C with anti-HKII antibody (1:100, Cell Signaling Technology, Cat#2867) or anti-COX subunit 1 antibody (1:200, AbCam Inc., Cat# ab14705). Sections were incubated with ABC Vectastain Kit according to the manufacturer's protocol, followed by incubation with DAB (Vector Labs). Mayer's hematoxylin was used to counterstain nuclei (Sigma-Aldrich).

### Clonogenic cell survival assay upon irradiation with or without PDK inhibition

Cells were plated in triplicates in a 6-well dish and grown for 24 hours. Cells were then treated with vehicle (negative control), 2mM or 4mM sodium dichloroacetate (DCA; Sigma) for another 24 hours, followed by irradiation using a Theratron Cobalt-60 machine to doses of 2, 4, and 6Gy at a dose rate of 1Gy/minute. Ten days post irradiation; cells were washed, fixed with ice-cold 70% EtOH, and stained with 1% Crystal Violet. Colony counting was performed using the Clono Counter software.

### Statistical analysis

IHC slides from HNSCC patients were evaluated by two pathologists. The slides were scored semi-quantitatively (0-3) for two criteria, intensity and distribution. Intensity was classified as 0: no staining, 1: weak, 2: moderate and 3: strong staining. Distribution was recorded as 0: no staining, 1: 1-20%, 2: 21-70% and 3: >=71 % of tumor cells with positive staining. Then, COX and HKII indices were calculated by multiplying intensity score by distribution score and COX/HKII intensity ratio and COX/HKII index ratio were computed accordingly. These ratios were also divided into three levels, >1: COX dominant, =1: Even, and <1: HKII dominant. Out of the 81 cases, no appropriate tumor sections were available for two cases and thus the final sample size includes 79 cases, 1 of which was not evaluable for HKII.

Differences in frequency distribution of each IHC marker by tumor characteristics, HPV and smoking status were tested by Pearson and Mantel-Haenszel chi-squares and Fisher's exact test where appropriate. Ordinal and binary logistic regression models were used to assess the effects of tumor HPV status, smoking, tumor location and their interactions on levels of IHC markers altogether or stratified by tumor location (tonsil vs. others). COX's proportional hazard models were employed to estimate hazard ratios (HR) and 95% confidence intervals (CI) for all deaths associated with selected IHC marker levels, tumor location and their interactions, stratified by radiation treatment history. These statistical analyses were performed by SAS version 9.3. In addition, one-way analysis of variance (ANOVA) and Newman-Keuls multiple range test (parametric) were used to compute and test mean ± s.d. of laboratory experimental groups. All statistical tests are 2-sided and nominal P values of .05 or less are regarded as significant.

## Abbreviations

CAIX: carbonic anhydrase IX
COX: cytochrome *c* oxidase
DCA: dichloroacetate
HIF1α: hypoxia-inducible factor 1α
HKII: hexokinase II
HNSCC: head and neck squamous cell carcinoma
HPV: human papilloma virus
OXPHOS: oxidative phosphorylation
PDK: pyruvate dehydrogenase kinase
TCA: tricarboxylic acid
